# Sucrose Monolaurate as a Stabilizer for Lactate Oxidase Electrodes at Low pH: A Structural Analysis Based on Grazing Incidence Small-Angle X‑ray Scattering

**DOI:** 10.1021/acs.langmuir.5c02857

**Published:** 2025-07-26

**Authors:** Isao Shitanda, Chiaki Sawahara, Noya Loew, Yuichi Takasaki, Taku Ogura, Hikari Watanabe, Masayuki Itagaki

**Affiliations:** † Department of Pure and Applied Chemistry, Faculty of Science and Technology, Tokyo University of Science, 2641 Yamazaki, Noda, Chiba 278-8510, Japan; ‡ Anton Paar Japan K. K., Riverside Sumida 1F, 1-19-9, Tsutsumi-dori, Sumida-ku, Tokyo 131-0034, Japan; § Research Institute for Science and Technology, 26413 Tokyo University of Science, 2641 Yamazaki, Noda, Chiba 278-8510, Japan

## Abstract

Sugars and sugar surfactants can increase the storage stability of enzyme electrodes. In this study, the feasibility of using sugar surfactants as stabilizers for enzyme electrode operation under acidic conditions was investigated along with their stabilizing mechanism. Lactate oxidase (LOx)–sucrose monolaurate-modified electrodes maintained ∼80% of their activity at pH 5.0, compared with ∼50% activity retention without a stabilizer. To elucidate the stabilizing mechanism, the structure of sucrose monolaurate with and without LOx on common electrode materials was analyzed using grazing incidence small-angle X-ray scattering (GI-SAXS). The results revealed that LOx was embedded in hexagonal arrangements and lamellar structures comprising sucrose monolaurate. Encapsulation protected the microenvironment of the enzyme against pH changes, without hindering its access to the substrate and mediator. This study confirms the high potential utility of GI-SAXS as a powerful tool for elucidating the structure-derived mechanisms of enzyme-electrode modifications.

## Graphical abstract

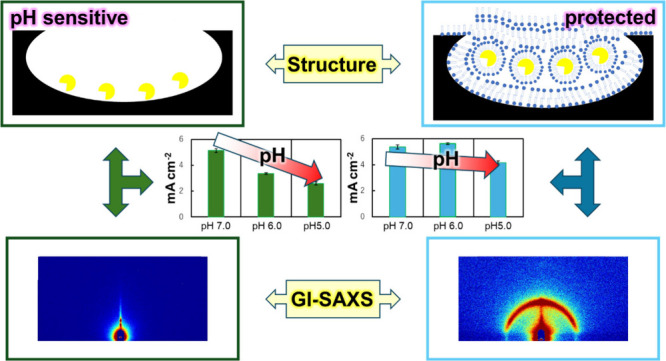

## 1 Introduction

Wearable enzymatic devices have attracted significant attention in recent years, and are expected to show high applicability in the fields of medicine and nursing care. Consequently, several studies have investigated noninvasive devices based on body fluids such as sweat, urine, tears, and saliva,^1–6^ many of which are electrochemical devices based on enzyme electrodes.

Au is a commonly used material in enzyme electrodes^7–10^ owing to high biocompatibility, excellent conductivity, easy surface modification using thiols, and a suitably large electrochemical window in most water-based solutions. Carbon, with excellent biocompatibility, good conductivity, reasonable surface modification possibilities, a suitably large electrochemical window in most water-based solutions, and abundant availability at low cost, is another commonly used electrode material.^9–12^ To date, several types of Au and C nanomaterials have been synthesized.

Among the various carbon materials, porous carbon is particularly popular for the immobilization of enzymes in electrochemical biosensors and biofuel cells.^10,12,13^ The performance of an enzyme electrode is determined by the reaction rate of the catalytic enzyme and the electron transfer rate between the enzyme and electrode, which is influenced by the choice of carbon material. MgO-templated mesoporous carbon (MgOC) is a commonly used porous carbon material.^14–18^ The pore size of MgOC can be effectively controlled by varying the size of the MgO template.^15^ Furthermore, the application of MgOC to enzyme electrodes enables stable enzyme loading, and the combination of mesopores and macropores permits the complete utilization of the three-dimensional space.^16,19^ To date, biosensors and biofuel cells have been fabricated using screen-printed MgOC for various applications, including wearable devices.^20–23^

A significant challenge for wearable biosensors, particularly those developed for monitoring metabolites in sweat, is stability in acidic conditions. While the pH of human sweat can be as low as 4.0,^24,25^ enzymes such as lactate oxidase (LOx) are most active and stable at neutral pH and lose their activity at acidic pH. The optimum pH of LOx, which depends on its origin, is generally pH 7.0 or higher.^26^ Several strategies for increasing the stability of LOx electrodes in acidic conditions, including the addition of a pH control layer^27^ or flux restricting layer^28^ and enzymatic encapsulation with hydrophobic carbon, have been proposed to date.^29^ These additions ensure a neutral-pH enzyme microenvironment.^30^

Another enzyme-microenvironment control strategy involves encapsulation with surfactants.^30^ However, the ability of surfactants to stabilize or destabilize enzymes varies with their type and concentration.^31,32^ Sugars, such as maltose or sucrose, are popular enzyme stabilizing agents. Sugar surfactants, such as sucrose monolaurate, can stabilize enzymes during the freeze–thawing and freeze-drying processes^33^ by facilitating the formation of amorphous matrices and interactions (hydrogen bonding) between proteins.

Therefore, in this study, sucrose monolaurate and other sugar surfactants are hypothesized to stabilize enzyme electrodes at acidic pH. On validation of this hypothesis, elucidating the structure of the sucrose monolaurate/enzyme layer immobilized on enzyme electrodes is vital for understanding the mechanism of the proposed stabilization.

Several techniques are used to elucidate the interactions between immobilized enzymes and their microenvironments, including surface plasmon resonance (SPR),^34^ surface enhanced Raman spectroscopy (SERS),^35^ atomic force microscopy (AFM),^36^ and attenuated total reflection Fourier-transform infrared spectroscopy (AT-FTIR).^37^ However, methods such as SERS, AFM, and AT-FTIR only yield morphological or chemical information on the utmost surface of the sample, and are unsuitable for the investigation of complex films.

Several studies have elucidated the structures of surfactant films using grazing incidence small-angle X-ray scattering (GI-SAXS).^38–43^ GI-SAXS is a variation of small-angle X-ray scattering (SAXS), which is commonly used to evaluate the structure of enzymes and surfactant conglomerates. Recent advances in laboratory-based SAXS devices have led to increased interest in this technique. Laboratory-based SAXS devices, now available from various companies, are independent of synchrotron availability; however, they typically require longer measurement times to compensate for the low X-ray intensity.^44–49^ Furthermore, the utilization of low-intensity X-ray reduces the risk of radiation damage to the sample.

For the elucidation of enzyme structures, SAXS and similar small-angle neutron scattering (SANS) techniques show high versatility in terms of the size and environment of enzymes.^48–50^ SAXS has been used to analyze the structures of various proteins in solution, including the detailed conformation of the nucleocapsid dimer of SARS-CoV-2,^46,51,52^ along with the structures of surfactants, including sugar surfactants, under various conditions.^53–55^

The interactions between proteins and surfactants have been investigated extensively by SAXS.^56–58^ Narayanan et al. studied the structure of the lysozyme–sodium dodecyl sulfate (SDS) complex in solution using SAXS and proposed a swelling micelle model reflecting the change in the electron scattering length density of the core and shell when the micelle interacts with the protein.^57^ Changes in the shape and slope of the scattering curves can be used to elucidate interactions between individual structural elements of surfactants and proteins. Such studies have been used to examine the influence of SDS and octaethylene glycol monododecyl ether (C_12_E_8_) on the unfolding and folding of proteins through the conversion of α-helices into β-sheets and vice versa.^59^ Kaspersen et al. investigated the refolding of SDS-unfolded globular proteins by the nonionic surfactants C_12_E_8_ and dodecyl maltoside (DDM).^60^ In most cases, C_12_E_8_ and DDM extract SDS from the SDS–protein complex without interacting directly with the protein.

GI-SAXS is used to characterize thin films and other surface nanostructures through analyses of the scattering pattern of an X-ray beam grazing the surface of the material at a low angle. Using this method, Tanaka et al. evaluated the structures of polyacrylate thin films^61^ and Shibuya et al. analyzed the mesoporous structure of mesoporous aluminum oxide films.^62^ Cortez et al. investigated polyelectrolyte–surfactant complex films on electrode materials in various set-ups.^38–41^ Moehl et al. analyzed the assembly of surfactants into a film using GI-SAXS.^42^ Mezei et al. elucidated the nanostructure of different surfactant–DNA complexes.^43^

This study investigates the structure of sucrose monolaurate thin films on common electrode materials using GI-SAXS along with the structural changes in these films in the presence of a coimmobilized enzyme, lactate oxidase (LOx), to elucidate the mechanism of the stabilizing effect of sugar surfactants on enzyme electrodes.

## 2 Materials and Methods

### 2.1 Electrochemical Measurements

Electrodes for electrochemical measurements were printed on Japanese paper using a screen printer (LS-150 TV, NewLong Co., Japan). First, five layers of carbon ink (JELCON CH-8, JUJO CHEMICAL CO., Japan) were printed and dried at 120 °C for 15 min. Next, two layers of MgOC ink were printed and dried overnight at 60 °C. The MgOC ink was fabricated by mixing MgOC (1 g) (Toyo Tanso Co. Ltd. Japan), polyvinylidene fluoride (7 mL) (KF #9305, Kureha Co., Japan) as a binder, and 1-methyl-2-pyrrolidone (4.8 mL) (Wako Pure Chemical Industries, Japan) as the solvent. The printed electrodes measured 1 cm^2^ (0.5 cm × 2 cm).

After treatment with UV-ozone for 12 min, the printed electrodes were modified by drop casting a 1,2-naphthoquinone (20 μL; 100 mM) (Kanto Chemical Co., Japan) solution in acetonitrile as a mediator followed by drying under reduced pressure. Next, a phosphate buffer solution (10 mM) containing LOx (40 U) (derived from *Enterococcus faecium*)^63^ was drop-cast onto the electrode and dried under reduced pressure. To manufacture electrodes with stabilizers, an enzyme solution containing either maltose (1 mM) (Tokyo Kasei Kogyo Co., Japan) or sucrose monolaurate (1 mM) (Sigma-Aldrich Co., USA), as indicated, was used.

Cyclic voltammetry was conducted in phosphate buffer (1 M) containing lactate (100 mM) at pH 7.0, 6.0, and 5.0 at a scan rate of 10 mV s^–1^ on an EmStat4S potentiostat (Palm Sens Co., The Netherlands). The fabricated LOx electrode, Pt wire, and a saturated KCl/silver/silver chloride electrode were used as the working electrode (WE), counter electrode (CE), and reference electrode (RE), respectively.

### 2.2 Fabrication of GI-SAXS Samples

Electrode-like substrates for GI-SAXS were fabricated on 3 cm-square glass plates. Au substrates were fabricated by masking the edges of the glass plates, retaining an empty 2 cm-square in the center, and vapor-depositing Au onto the glass. Carbon substrates were synthesized by masking the edges of the glass plates, retaining an empty 2 cm-square in the center, applying a thin layer of carbon ink with a squeegee, and drying at 120 °C for 15 min. MgOC substrates were fabricated by manually screen-printing MgOC ink onto carbon substrates, and drying at 60 °C for 12 h. The same carbon and MgOC inks were used as electrodes for electrochemical measurements.

Four samples, namely, blank, LOx-modified, sucrose monolaurate-modified, and LOx-sucrose monolaurate-*co*-modified, were prepared for each type of substrate. Before modification, the Au substrates were cleaned by ultrasonication in NaOH­(aq) (0.5 M) and rinsed with deionized water. The cleaned Au substrates were modified by drop casting or spin coating. An SC-200 (Oshigane Co., Japan) was used for spin coating at 1000 rpm for 30 s. After cleaning by UV-ozone irradiation for 12 min, the carbon and MgOC substrates were modified by drop casting and dried under reduced pressure for 90 min. The amount per unit area and concentration of the modification solution in each case were equivalent to those used to modify electrodes for electrochemical measurements.

### 2.3 GI-SAXS Measurements

A laboratory-based SAXS system SAXSPoint 5.0; Anton Paar GmbH, Austria) was used for GI-SAXS with a Cu Kα X-ray source. In general, scattering patterns were acquired over 5 min at a detector distance of 600 mm. The scattering patterns for sucrose monolaurate- and LOx-sucrose monolaurate-modified substrates were acquired over 20 min at a detector distance of 400 mm.

### 2.4 SAXS Measurements of Sucrose Monolaurate Solutions

A laboratory-based SAXS system (Xeuss 3.0; Xenocs SAS, France) with a Cu K-α X-ray source was used for the SAXS analysis of sucrose monolaurate solutions (1.0, 5.0, 10, and 20 mM) in phosphate buffer (10 mM). Data were acquired over 900 s at a detector distance of 600 mm.

### 2.5 Contact Angle Measurements

Contact angles were measured on cleaned blank substrates using water and a contact angle meter (DM 300, Kyowa Interface Science Co., Japan).

## 3 Results and Discussion

### 3.1 Electrochemical Evaluation of Stabilizers for LOx Electrodes

LOx electrodes containing maltose and sucrose monolayers were tested for their stability under acidic conditions (Figure 1). As expected, without additives, the response current, quantified from the oxidation peak current density, decreased with decreasing pH. At neutral pH, the shape of the cyclic voltammograms did not change significantly on stabilizer addition (Figure 1), indicating that the stabilizer did not affect the formation or reaction of the electric double layer. Furthermore, the response current densities at pH 7.0 with and without the stabilizer were similar (Figure 1), indicating that the stabilizer did not directly affect the enzyme reaction.

**Figure 1:**
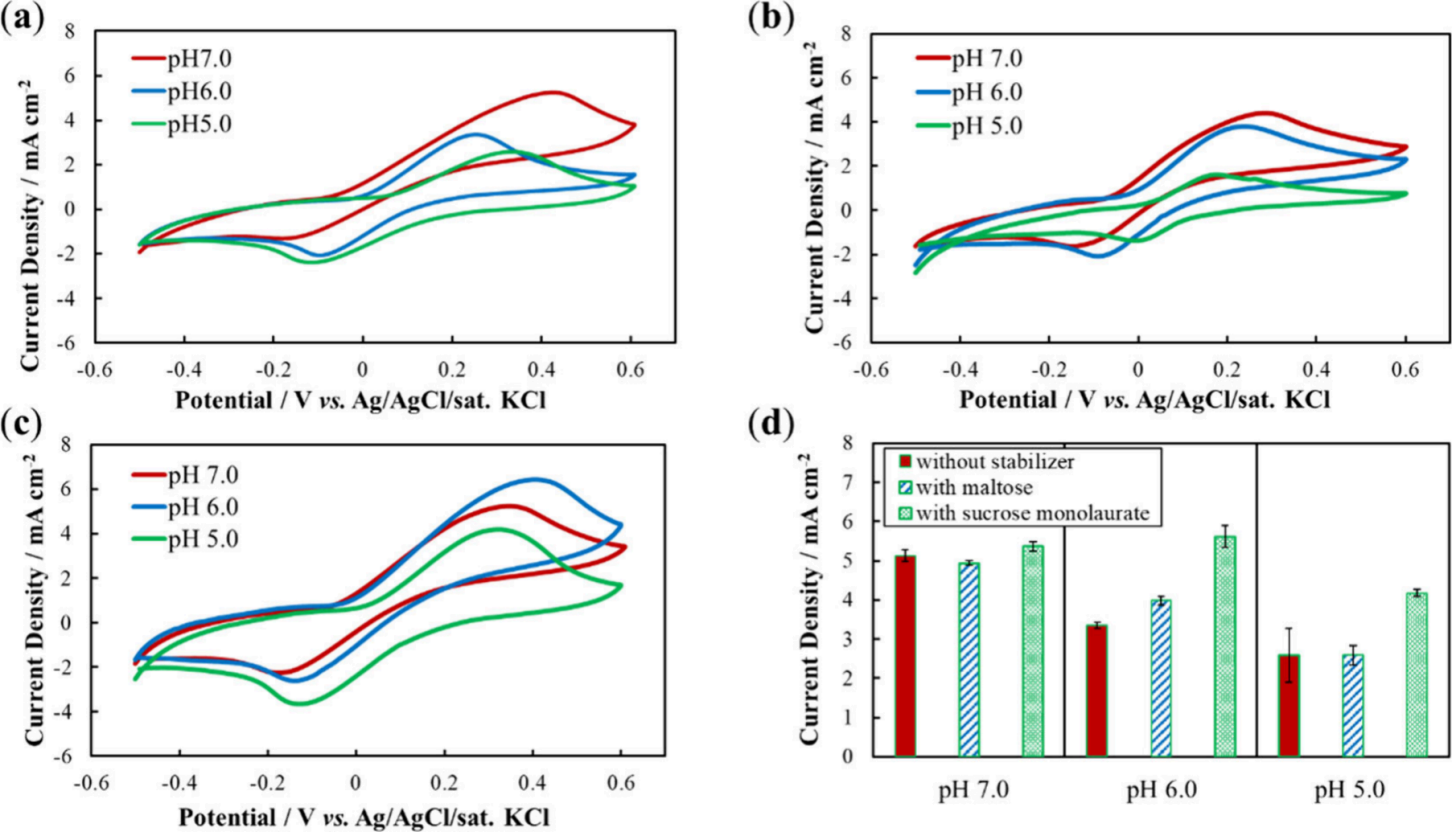
Cyclic voltammetry of LOx electrodes with stabilizers at different pH values with lactate (100 mM) in phosphate buffer (1 M) at pH 7.0, 6.0, and 5.0 for electrodes fabricated (a) without stabilizer, (b) with maltose, and (c) with sucrose monolaurate. (d) Comparison of oxidation peak current densities.

Maltose is reported to stabilize the structure and activity of dried enzymes, thereby increasing the shelf life of enzyme electrodes. Here, maltose was used as the stabilizer under acidic conditions. With maltose, the response current at pH 6.0 was lower than that at pH 7.0, but higher than that at pH 6.0 without a stabilizer (Figure 1d). At pH 5.0, the response currents without stabilizers and with maltose were similar. Therefore, as a stabilizer under acidic conditions, maltose was somewhat effective at pH 6.0 and ineffective at pH 5.0.

Notably, with sucrose monolaurate, the response current at pH 6.0 was similar to that at pH 7.0 (Figure 1d). Furthermore, the response current at pH 5.0 was ∼ 80% that at pH 7.0, whereas, without a stabilizer or with maltose, the response decreased to 50% (Figure 1d). These results indicate that sucrose monolaurate is an effective stabilizer for LOx electrodes under acidic conditions.

### 3.2 GI-SAXS Analysis of LOx and Sucrose Monolaurate Films on Various Surfaces

Surfactants such as sucrose monolaurate are likely to form very different layers on hydrophilic and hydrophobic surfaces; therefore, the contact angles of sucrose monolaurate were measured for each substrate. All substrates were hydrophilic; water contact angles of 75.5, 76.6, and 70.4° were observed for Au, carbon, and MgOC, respectively.

The two-dimensional GI-SAXS scattering patterns obtained for various substrates are shown in Figure 2–5. Au substrates showed intense out-of-plane peaks owing to the flat surface and high reflectivity of vapor-deposited Au (Figure 2). The mesoporous nature of the MgOC substrate was reflected in its scattering pattern, which contained a high-intensity center in both the out-of-plane and in-plane directions (Figure 5) caused by the scattering of the incident X-ray beam in random directions by the rough surface of the substrate. The carbon substrate exhibited an intermediate substrate-derived pattern congruent with the intermediate surface roughness of carbon (Figure 4).

**Figure 2:**
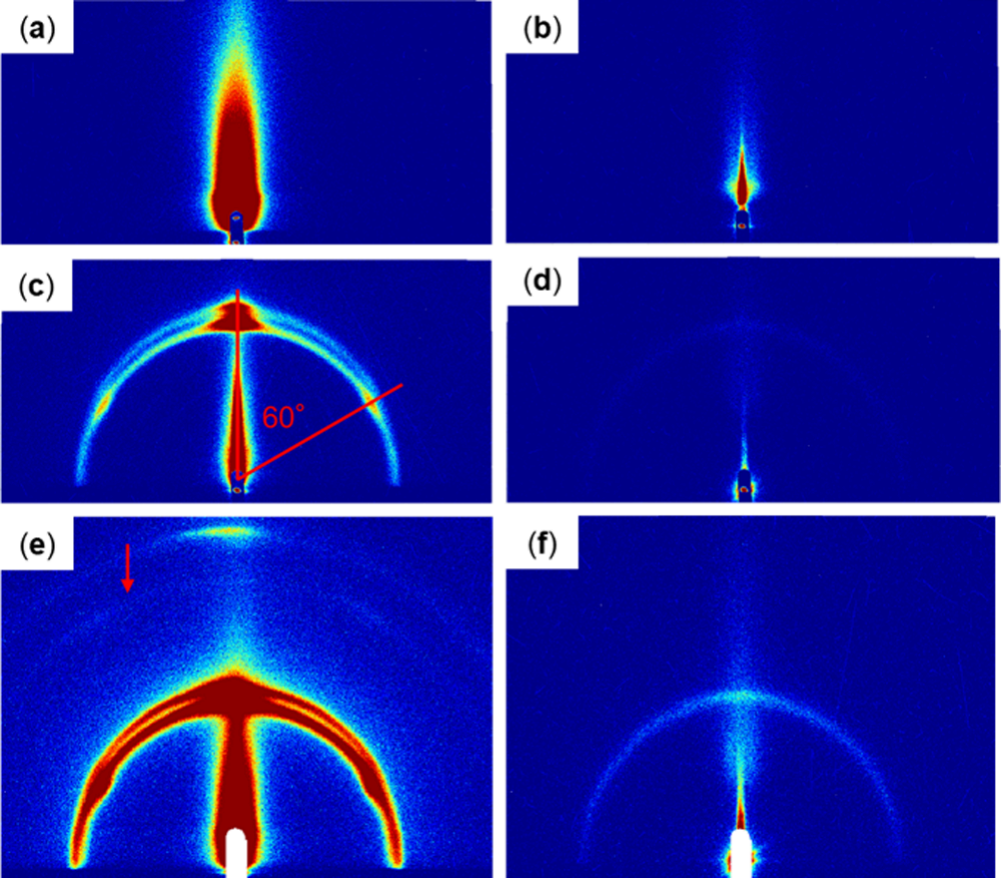
GI-SAXS scattering patterns of Au substrates with the following drop-casting modifications: (a) blank, (b) LOx, (c, e) sucrose monolaurate, and (d, f) LOx and sucrose monolaurate. Patterns acquired over (a–d) 5 min and (e, f) 20 min.

**Figure 3:**
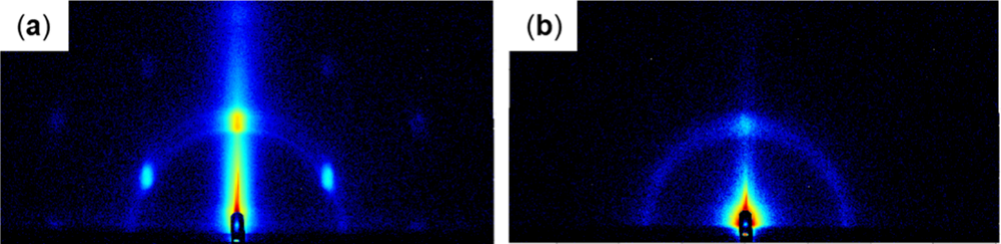
GI-SAXS scattering patterns of Au substrates with the following spin-coating modifications: (a) sucrose monolaurate as well as (b) LOx and sucrose monolaurate. Patterns acquired over 20 min.

**Figure 4:**
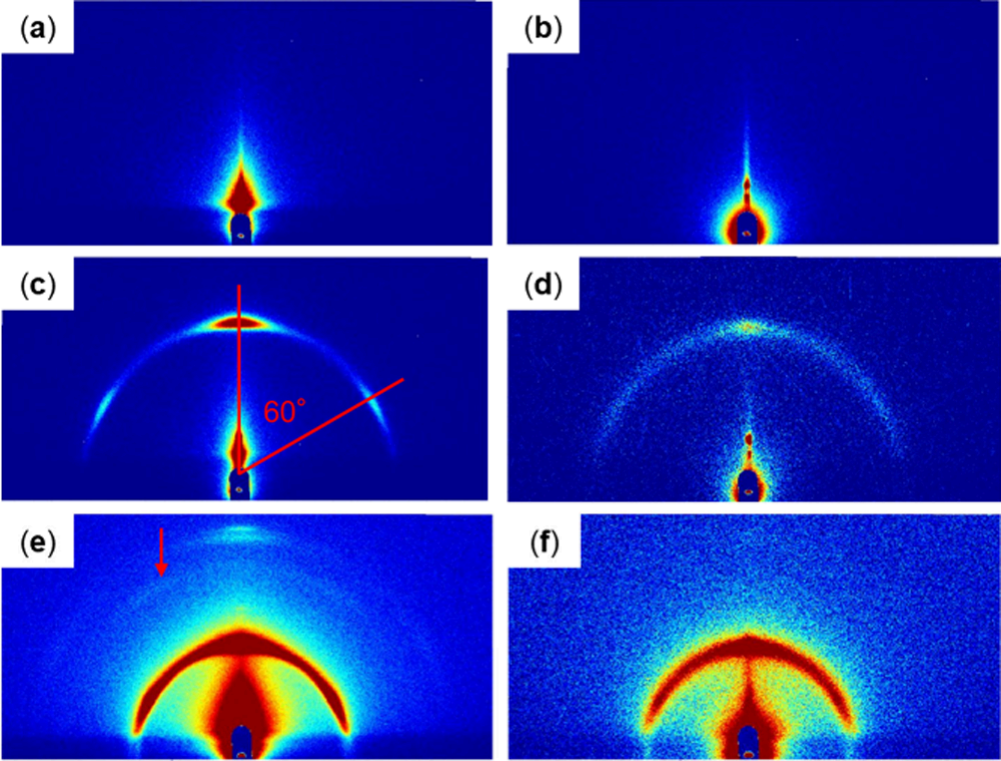
GI-SAXS scattering patterns of the following carbon substrates: (a) blank and modified with (b) LOx, (c, e) sucrose monolaurate, and (d, f) LOx and sucrose monolaurate. Patterns acquired over (a–d) 5 min and (e, f) 20 min.

**Figure 5:**
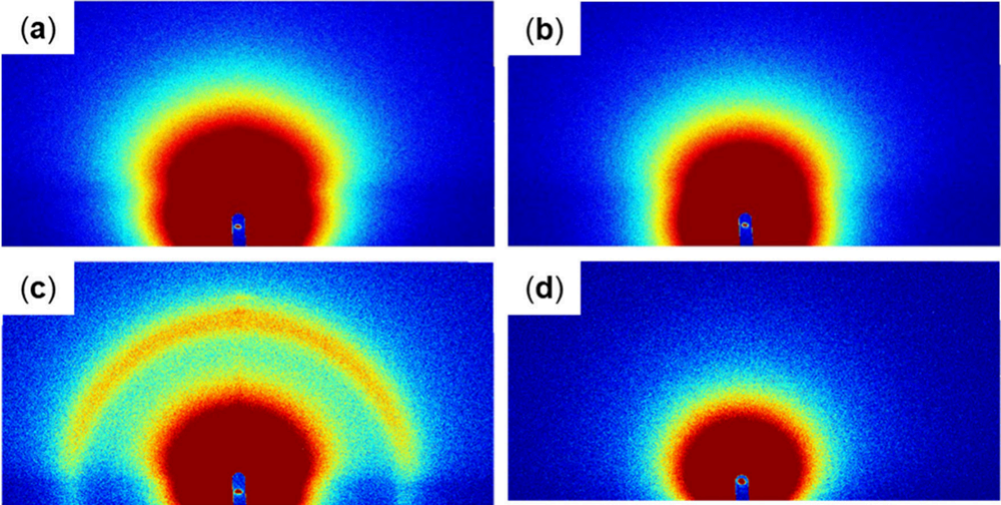
GI-SAXS scattering patterns of the following MgOC substrates: (a) blank and modified with (b) LOx, (c) sucrose monolaurate, and (d) LOx and sucrose monolaurate. Patterns acquired over (a, b) 5 min and (c, d) 20 min.

In all cases, the substrate-derived signals weakened to varying degrees in the modified samples. LOx-modified substrates showed only weakened substrate-derived patterns with no distinctive patterns owing to the enzymes (Figures 2, 4, and 5), indicating randomly oriented LOx on the surface that does not form discernible structures. Furthermore, in contrast to the clear results of transmission-type SAXS, the intramolecular structure of LOx appeared invisible in GI-SAXS, possibly because significantly more LOx molecules are expected to be in the path of X-rays in a typical transmission-type SAXS than in GI-SAXS.

All sucrose monolaurate-modified substrates showed at least one semicircular pattern (Figures 2–5). Secondary semicircular patterns were observed with Au and carbon substrates, which became clearer when acquired over a prolonged duration (Figure s[Fig fig2]e and 4e); these semicircular patterns exhibited high-intensity subpatterns at 60° (Figures 2c and 4c). On modifying Au substrates with sucrose monolaurate by spin coating, increased-intensity localized patterns were observed at 60° angles along with a semicircular pattern with a very low intensity (Figure 3a). Spin coating leads to a thinner surface modification layer than drop casting. Therefore, the results obtained with spin-coated Au substrates are expected to represent the structure of sucrose monolaurate on Au.

On comodification with both LOx and sucrose monolaurate, the patterns of substrates were significantly weaker than the pattern of sucrose monolaurate samples, and sometimes disappeared completely (Figures 2–5). With MgOC substrates, the comodified samples showed no discernible modification-derived patterns (Figure 5d). With Au and carbon substrates, the localized patterns at 60° angles disappeared and only semicircular patterns remained (Figures 2d,f, 3b, and 4d, and 4f). Furthermore, a weak semicircular pattern was observed for Au substrates modified by drop casting (Figure 2d,f).

According to the literature, semicircular scattering patterns are commonly caused by lamellar structures with random directional axes.^45^ Localized patterns at 60° angles indicate either an ordered lamellar structure at a 60° angle to the substrate or a hexagonal structure. Ordered, angled-lamellar, and hexagonal structures differ in the position of the secondary, tertiary, and other intensity peaks relative to the position of the primary peak. To quantify the positions of the intensity peaks, two-dimensional scattering patterns were converted into one-dimensional scattering profiles (Figure 6). For sucrose monolaurate-modified Au and carbon substrates, peaks were observed at 1.72, 2.96, and 3.50 nm^–1^ (Figure 6a–c), consistent with the Bragg peak positions of hexagonal structures, which show a 1:3^1/2^:2 ratio for the *q* values of primary, secondary, and tertiary peaks.^64^ For spin-coated Au substrates, low-intensity secondary and tertiary peaks were observed along with an extremely intense primary peak (Figure 6b). In LOx–sucrose monolaurate comodified samples, the primary peak broadened, decreased in intensity, and shifted slightly to lower *q* values, whereas the secondary and tertiary peaks disappeared (Figure 6a–c). Furthermore, with the MgOC substrate, the primary peak was broad and weak in the spectrum of sucrose monolaurate-modified samples and disappeared in the spectrum of LOx–sucrose monolaurate-modified samples (Figure 6d).

**Figure 6:**
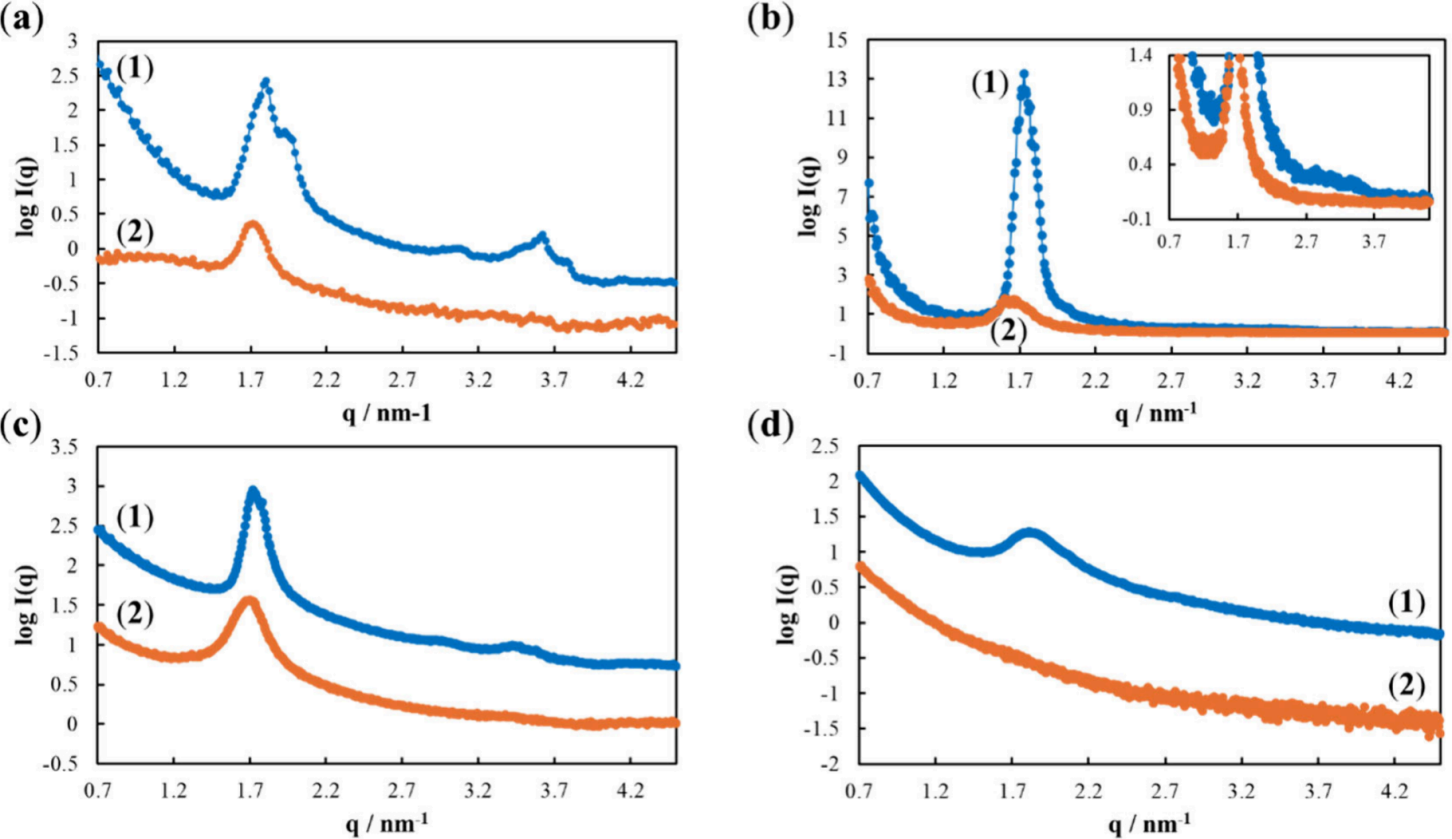
Averaged GI-SAXS scattering intensity for the following substrates: (a) Au (drop-cast), (b) Au (spin-coated) [inset: zoomed in images], (c) carbon, and (d) porous carbon modified with (1) sucrose monolaurate and (2) LOx and sucrose monolaurate.

These results suggest that sucrose monolaurate exists as hexagonal structures and as lamellar structures with random directional axes on the substrate. In the presence of LOx, the structures become disordered, causing the peaks to shift toward the left, broaden, and lose their intensity. Hexagonal structures may be more affected than lamellar structures. These effects became less detectable with increasing substrate-surface roughness. Owing to the complex surface structure of MgOC, no hexagonal or lamellar structures were observed when MgOC substrates were modified with sucrose monolaurate. In the presence of LOx, the modification-derived scattering signals were too weak to be observed. The carbon substrate presumably shows a rougher surface than the Au substrate but a significantly smoother surface than the MgOC substrate. Accordingly, the scattering signals of the carbon substrate were less clear than those of the Au substrate.

Furthermore, the results for spin-coated Au substrates suggest that the first layer of sucrose monolaurate predominantly comprises hexagonal structures; subsequent layers are more lamellar in nature. Because these lamellar structures are formed on top of hexagonal structures, they are formed with random directional axes.

### 3.3 SAXS Analysis of Sucrose Monolaurate in Solution

The transmission-type SAXS of increasing concentrations of sucrose monolaurate solutions was used to examine the possible structural changes in sucrose monolaurate during drying on the substrate (Figure 7). Because the critical micellar concentration of sucrose monolaurate is 0.3 mM,^65^ micelle formation is expected at all measured concentrations. As expected, the shapes of the scattering profiles for sucrose monolaurate solutions with concentrations of 1 and 5 mM are consistent with the profiles of core–shell surfactant micelles containing alkyl groups with a greater electron density than the core and surrounding solvent.^66^ Distance distribution functions, *P*(*r*), calculated using indirect Fourier transformation were used to determine the maximal particle diameters (*D*_max_) of the micelles. The diameter of the micelles (*D*_max_) increased with increasing sucrose monolaurate concentration (from 6.0 nm at 1 mM to 17 nm at 5 mM).

**Figure 7:**
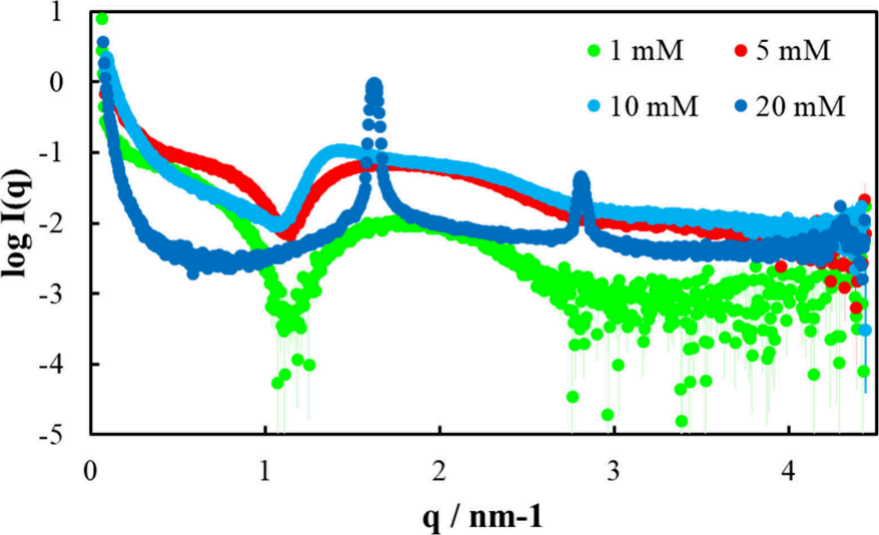
SAXS scattering intensity of sucrose monolaurate solutions (1.0, 5.0, 10, and 20 mM) in phosphate buffer (10 mM).

With 10 mM sucrose monolaurate, a decrease in the scattering-profile slope was observed over the *q*-range of 0.25–1 nm^–1^. Usually, a decrease in the slope in this range indicates a structural change from spherical to plate-like.^67^ At 20 mM, two strong peaks with *q* values of 1.63 and 2.81 nm^–1^ were observed; the *q* value of the second peak was ∼3^1/2^ times that of the first peak, indicating the formation of hexagonal-structure sucrose monolaurate at high concentrations.

These results indicate that hexagonal-structure sucrose monolaurate is formed when the modification solution evaporates, becoming more concentrated.

### 3.4 LOx Electrodes Containing Sucrose Monolaurate

Schematics of the structure of LOx-sucrose monolaurate films on Au and MgOC electrodes are shown in Figure 8. According to the literature, surfactants coat hydrophilic surfaces with their hydrophilic heads toward the surface. As mentioned previously, the results of contact angle measurements indicate a hydrophilic surface on Au, carbon, and MgOC. Notably, mediator-modified MgOC electrodes exhibit a higher wettability than unmodified electrodes, indicating a hydrophilic surface on the mediator-modified MgOC electrodes. As expected, the detailed views in Figure 8b–d show electrode surfaces coated with a layer of surfactant with the hydrophilic heads toward the surface. The results of this study indicate that sucrose monolaurate forms hexagonal structures close to the electrode and lamellar structures with random directional axes farther away. In Figure 8a, the hexagonal and lamellar structures are depicted as densely packed circles and stacked wavy lines, respectively. Hexagonal-structure sucrose monolaurate is also present in high-concentration solutions. Micellar core–shell sucrose monolaurate species found in low-concentration solutions likely merge and form rod-like structures, which subsequently assemble into hexagonal aggregates. Cross-sections of the assembled rod-like structures extending in the out-of-plane direction are shown in Figure 8b. With increasing deposition and aggregation as well as concentration enhancement, these rod-like structures merged into lamellar structures. Notably, hexagonal structures were not observed on MgOC substrates, possibly because rod-like sucrose monolaurate structures are predominantly present within the pores of MgOC, and are either hidden from the X-ray beam or not assembled into hexagonal structures. Cross-sections of rod-like structures buried in a carbon pore are shown in Figure 8d.

**Figure 8:**
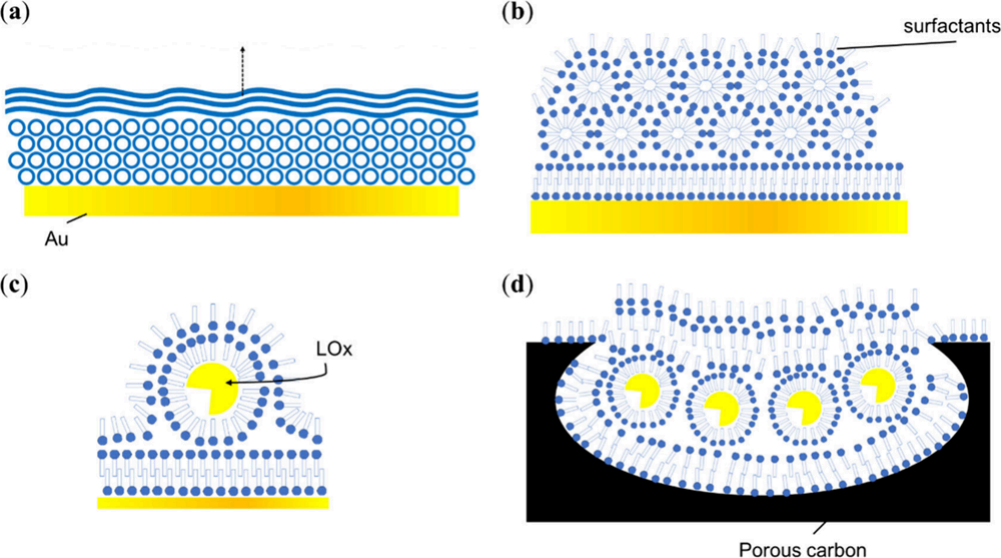
Schematics of sucrose monolaurate with LOx on an (a–c) Au and (d) MgOC electrode. (a) Overview with hexagonal and lamellar structures. Detailed views of (b) the electrode surface, (c) encapsulated LOx, and (d) a carbon pore.

Proteins and surfactants form a variety of complex structures depending on their nature and environment.^30,31,57^ Electrochemical measurements showed that LOx maintained its activity; consequently, the structure of LOx–sucrose monolaurate-modified electrodes were also maintained (Figure 1). Furthermore, sucrose monolaurate appeared to protect the structure of LOx under acidic conditions (Figure 1). GI-SAXS indicated that the hexagonal structures of sucrose monolaurate became undetectable in the presence of LOx, while the lamellar structures became less defined, i.e., the primary peak broadened and lost its intensity (Figure 6). Moreover, the primary peak shifted to slightly lower *q* values, indicating an increase in the average layer thickness.

These results confirm the incorporation of LOx into the rod-like structures and between the lamellar layers of sucrose monolaurate (Figure 8c,d). The rod-like structures may be more likely to merge into lamellar structures when they contain LOx. Additionally, the rod-like structures containing LOx are likely to comprise an uneven cross-section, i.e., they are likely to resemble rods with bulges. The combination of rods with and without LOx is likely to result in an assembly of rods with different diameters. In contrast to hexagonal aggregation in the absence of LOx, all these possibilities lead to a relatively random aggregation of rod-like structures.

The incorporation of LOx into the sucrose monolaurate layers described here is in accordance with sucrose monolaurate encapsulating LOx in solution and forming core–shell micelles, which are one of the complex structures formed by proteins and surfactants.^31,32,58^

Additionally, the encapsulation of LOx by sucrose monolaurate explains the increased stability of the LOx-sucrose monolaurate electrode in acidic conditions observed in this study. Surfactant layers separate and compartmentalize water-based solutions. The ability of a molecule to cross the surfactant layer depends on its size and charge. Although phospholipid layers, which comprise the majority of cell membranes, are technically not surfactants, several studies have extensively investigated molecules that can cross these layers. Studies on cell membranes indicate that protons and phosphate ions do not cross this membrane, whereas water, lactate, and quinones do. Therefore, in LOx–sucrose monolaurate-modified electrodes, sucrose monolaurate does not significantly affect the structure of LOx, movement of water, or diffusion of the substrate or mediator. However, when LOx is encapsulated by sucrose monolaurate, the pH of its microenvironment is similar to that at the time of electrode modification and is not easily affected by the pH of the bulk during operation because protons do not cross the encapsulating layers and are aided by phosphate buffer ions from the modification solution trapped inside the system. Therefore, LOx–sucrose monolaurate-modified electrodes and LOx-modified electrodes behave similarly at neutral pH, whereas LOx–sucrose monolaurate-modified electrodes show higher activity at acidic pH.

## 4 Conclusion

In this study, the sugar surfactant sucrose monolaurate was evaluated as a stabilizer for LOx electrodes under acidic conditions focusing on the structural aspects of the stabilizing mechanism. The structures of sucrose monolaurate with and without LOx on the electrode materials were elucidated using GI-SAXS. Sucrose monolaurate core–shell micelles were first deposited in rod-like structures, which subsequently assembled into hexagonal arrangements. Further from the electrode surface, sucrose monolaurate formed lamellar structures with random directional axes. In LOx–sucrose monolaurate-modified electrodes, LOx is embedded into these structures, which reduces their regularity, resulting in a reduction in the scattering intensity. Encapsulated sucrose monolaurate protects the microenvironment of LOx against pH changes while enhancing access to the substrate and mediator. Therefore, compared with an activity retention of ∼ 50% without a stabilizer, LOx–sucrose monolaurate-modified electrodes maintain ∼80% of their activity at pH 5.0.

The results of this study confirm that GI-SAXS is a powerful tool for elucidating the mechanism through which stabilizers function in enzyme electrodes. Determining this mechanism could facilitate the design and development of new and improved stabilizers, which, in turn, could lead to the development of highly stable enzyme electrodes and high-performance biodevices.

## Abbreviations

CCR2: CC chemokine receptor 2
CCL2: CC chemokine ligand 2
CCR5: CC chemokine receptor 5
TLC: thin layer chromatography
